# Stigma and Social Avoidance in Adults with Essential Tremor

**DOI:** 10.1002/mdc3.13774

**Published:** 2023-06-21

**Authors:** Padraig O'Suilleabhain, Diane S. Berry, Duane A. Lundervold, Travis H. Turner, Madeline Tovar, Elan D. Louis

**Affiliations:** ^1^ Department of Neurology University of Texas Southwestern Medical Center Dallas Texas USA; ^2^ Department of Psychological Science University of Central Missouri Warrensburg Missouri USA; ^3^ Department of Neurology Medical University of South Carolina Charleston South Carolina USA; ^4^ Medical School, University of Texas Southwestern Medical Center Dallas Texas USA

**Keywords:** essential tremor, stigma, psychological distress, social avoidance

## Abstract

**Background:**

People with essential tremor (ET) can be subject to stigma, and some adopt avoidance behaviors. Characteristics associated with ET stigma and the relationship between perceived stigma and social dysfunction have not been studied.

**Objectives:**

To discern predictors of perceived stigma and social dysfunction in ET, and to identify potentially treatable psychological factors associated with social dysfunction.

**Methods:**

We surveyed ET patients (*n* = 158) on recalled stigma incidents and social dysfunction related to tremor, as well as clinical and demographic characteristics including tremor severity, and psychological constructs including anxiety, depression, mindfulness, resilience, and narcissism.

**Results:**

Worse tremor severity (Standardized beta [SB] 1.4, *P* < 0.001) especially among younger participants (interaction of age and tremor severity SB −0.9, *P* < 0.001) and presence of vocal tremor (SB 0.7, *P* = 0.002) predict perceived stigma. 53/157 (33.8%) participants met criteria for social dysfunction, employing maladaptive avoidance strategies. Scores for perceived stigma (Odds Ratio [OR] 1.2, *P* = 0.002), depression (OR 1.5, *P* = 0.004) and stigma psychological distress (OR 1.2, *P* = 0.001) as well as sex (OR 4.3 for females, *P* = 0.045) predicted social dysfunction.

**Conclusions:**

Depression and stigma psychological distress contribute to social dysfunction related to ET stigma. Treating these psychological factors may mitigate social avoidance behaviors prevalent among susceptible individuals: those who most perceive ET stigma, i.e. relatively younger patients with worse tremor or with vocal tremor, and in particular females who are more prone to social dysfunction than males with the same degree of perceived stigma.

Involuntary movements such as tremor can attract unwanted attention and judgment from observers. Essential tremor (ET), for example, is not well understood by the public and can give rise to impressions of nervousness, frailty or substance abuse.^1^ We recently developed a new tool for studying such negative attributions—or stigma—associated with ET.^2^ This quantifies three dimensions of ET‐related stigma: perceived or experienced stigma ie, an individual's recollection of subjective ET stigma events, and the psychological distress and social dysfunction attributed to ET stigma. Our previous research found most ET patients perceived stigma and a substantial minority reported social dysfunction.^2^ The first objective of the present report was to identify factors predictive of ET stigma. We hypothesized worse manual difficulty and presence of vocal tremor predict perceived stigma and thus social dysfunction, and explored additional clinical and demographic factors as possible contributing factors. The second objective was to understand the relationship between perceived stigma and social dysfunction. We hypothesized those who perceive more stigma would manifest more social dysfunction, and explored a range of psychological constructs as possible contributing factors, hoping to identify one or more treatable constructs with potential to reduce social dysfunction due to ET stigma.

## Methods

### Participants

Our sample consisted of ET patients who took part in our previously reported validation study of our measure of ET stigma.^2^ These cases were identified from a search of the electronic medical records of patients attending a movement disorders clinic at the University of Texas Southwestern Medical Center. Search criteria included: (1) diagnosis of ET at a clinic visit within the prior 2 years; (2) 18–80 years of age; (3) no diagnosis of dementia or parkinsonism; and (4) a working email address supplied in the electronic medical record. The study was approved by the University of Texas Southwestern Medical Center Institutional Review Board. An information sheet explaining the study was emailed to eligible patients. Those agreeing to participate followed a link to a series of electronic questionnaires.

### Stigma Outcomes

Perceived stigma is measured via survey estimation of recalled lifetime subjective experiences of ET stigma. Response options range from 0 (never) to 4 (very often/> 100 times) for 13 different types of ET stigma that they may have encountered (eg, thought to be more nervous or anxious than was really the case, assumed to be less physically capable than was really the case, thought to have overused alcohol or drugs). Responses to the 13 items are summed, yielding a total score that ranged from 0 to 52.^2^

Social dysfunction related to ET stigma is determined via survey assessing the frequency with which people employ the following five social strategies in response to peoples’ reactions to their tremor: (1) keeping a low profile in group settings; (2) using extra alcohol before interacting with people; (3) avoiding volunteering with others; (4) avoiding social events; and (5) avoiding participating in sports or games with others. This survey was taken twice by most participants across an interval of 1 month, and the average is used for this analysis. Scores of 2 or higher correspond to frequent use of at least one of the avoidance strategies queried or occasional use of two or more; this is the criterion for social dysfunction in this analysis, as it is the threshold at which people are interested in an intervention.^2^

### Clinical‐Demographic Characteristics

Tremor severity is measured with the Tremor Disability Scale—Revised (TREDS‐R)^3^ which captures subjective difficulty performing 20 manual tasks, with total score ranging from 20 (no difficulty with any of the tasks) to 80 (unable to perform any of the tasks).

Subjects also reported presence of head tremor; presence of vocal tremor; presence of another health condition said by the participant to be a bigger problem than ET; sex; occupational status; current age; and age at onset of tremor; from which was calculated duration living with tremor.

### Tremor Treatments

Subjects reported treatment with three medications: propranolol, primidone and topiramate with response options current use, past use, never used, do not recall. Treatment with deep brain stimulation was also reported.

### Psychological Constructs

#### ET Stigma Psychological Distress

Respondents indicate the extent to which they endorse negative emotions (eg, embarrassment, anger, frustration) in relation to tremor. Response options range from 0 (strong disagreement) to 4 (strong agreement), yielding a total score ranging from 0 to 44.^2^

#### Mindfulness

The Five Facets of Mindfulness Questionnaire has a range from 0 to 195, with higher scores reflecting greater mindfulness ie, observing, describing, acting with awareness, non‐judging of inner experience, and non‐reactivity to internal experience.^4^

#### Anxiety

The Hospital Anxiety & Depression Scale—Anxiety has seven items with a range from 0 to 21 at the highest level of anxiety.^5^

#### Depression

The Hospital Anxiety & Depression Scale—Depression has seven items with a range from 0 to 21 at the highest level of depression.^5^

#### Cognitive Fusion

The Cognitive Fusion Questionnaire measures the extent to which a person actions are fused to their thoughts. The score range is from seven which represents maximum cognitive defusion in which thoughts can be seen as mental events not needing to be acted on, up to 49 representing greatest difficulty disentangling one's thoughts from one's behaviors.^6^

#### Acceptance & Action

The Acceptance & Action Questionnaire is a measure of psychological flexibility with seven items. The score range is from seven for a high level of psychological flexibility, to 49 representing a low flexibility such that feelings and memories get in the way of a person's pursuit of success and happiness.^7^

#### Commitment to Action

The Committed Action Questionnaire has eight items with total score from 0 when uncommitted to action to 48 when highly committed to action. “It represents an individual's general propensity to persist in goal‐directed behavior.”^8^

#### Locus of Control

The Rotter's Locus of Control Scale has 29 items with total score from 0 corresponding to the person's belief that control over the outcome of events resides within the person, to 29 corresponding to belief that events are due to external forces beyond the person's influence.^9^

#### Resilience

The Connor Davidson Resilience Scale total score ranges from 0 representing low resilience to 100, the maximum score for psychological resilience.^10^

#### Narcissism

The Narcissistic Personality Inventory has 40 items with total score from 0 to 40, “the high NPI scorer … [is] relatively dominant, extraverted, exhibitionistic, aggressive, impulsive, self‐centered, subjectively self‐satisfied, self‐indulgent, and non‐conforming.”^11^

#### Self‐Monitoring

The Self‐Monitoring Scale has a range from 0 to 25, with higher scores reflecting greater self‐monitoring ie, consciously using impression‐management strategies in interactions, by adjusting behavior to the situation and through attention to the signals being sent to others.^12^

### Statistical Analyses

Associations of perceived stigma and social dysfunction were initially analyzed with univariate tests. Because perceived stigma score was not normally distributed, it was analyzed with Wilcoxon rank sum test for categorical independent variables with two levels, test of equality of medians for multi‐level categories, and linear regression for continuous independent variables. For social dysfunction, Fisher's exact test was used for categorical independent variables and simple logistic regression for continuous ones. Perceived stigma was then modeled in relation to tremor severity using multiple linear regression, adding clinical‐demographic characteristics as predictors and as potential interaction terms with tremor severity. Social dysfunction was modeled in relation to perceived stigma using logistic regression, adding clinical‐demographic characteristics and psychological constructs.

## Results

Of 862 eligible patients, 158 (18.3%) enrolled and completed most or all questionnaires. Distributions of demographic and clinical characteristics of participants are shown in Table 1. Median age was 70, with range was 30–80 years and interquartile limits 63–74 years.

**TABLE 1:** Distribution of demographic, clinical and psychological variables

Variable	Distribution
Demographics	
Sex (female)	82 (51.9%)
Current age	66.7 (10.4), 70
Employment status: Working full time	43 (27.2%)
Working part time	12 (7.6%)
Not working	88 (55.7%)
Disabled	15 (9.5%)
Clinical measures	
Tremor disability scale	34.1 (10.2), 34
Age of tremor onset	44.4 (18.5), 48
Duration of tremor^a^	22.6 (17.7), 17.5
Vocal tremor (present)	44 (27.9%)
Head tremor (present)	59 (37.3%)
Bigger health concern (present)	73 (46.2%)
Psychological contructs	
Social dysfunction (present)	53 (33.8%)
Perceived stigma	12.9 (11.0), 10
Stigma psychological distress	17.3 (11.4), 17
Depression	3.7 (3.6), 3
Anxiety	4.8 (4.4), 4
Mindfulness	138.9 (21.3)
Commitment to action	34.4 (8.0)
Locus of control	11.0 (3.9)
Acceptance and action	15.7 (9.4), 13
Cognitive fusion	17.1 (10.7), 14
Resilience	77.6 (14.6), 78
Narcissism	11.5 (6.3)
Self‐monitoring	9.4 (3.8)

*Note*: Sample *N* = 158. For normally distributed continuous variables, values reflect mean (SD). For continuous variables failing the Shapiro Wilk test values indicate mean (SD), median. For categorical variables, values represent *N* (%).

^a^ Duration, current age minus age of tremor onset.

### Perceived Stigma

153/158 (96.8%) participants had perceived one or more lifetime experiences of ET stigma. Median score was 10 with range 0–49 and interquartile limits 4–17.75. As hypothesized, worse tremor severity was associated with greater perceived stigma, with regression coefficient *β* = 0.45, *P* < 0.001. Other clinical‐demographic characteristics predictive of perceived stigma on univariate analysis (Table 2) were younger current age, earlier age of onset, longer duration with tremor, disabled occupational status, and presence of vocal tremor. Nine of the 11 psychological constructs were associated with perceived stigma. In the best multiple regression model with clinical‐demographic characteristics as independent variables, perceived stigma is predicted by tremor severity (standardized beta [SB] 1.4, *P* = <0.001), vocal tremor (SB = 0.7, *P* = 0.002), and by two terms interacting with tremor severity: age (SB = −0.9, *P* < 0.001) and vocal tremor (SB = −0.6, *P* = 0.01); model *F* (4,144) = 20.3 and *P* < 0.0001. Participants who never used the tremor treatments tended to have lower perceived stigma, reaching statistical significance for primidone only (Table 3).

**TABLE 2:** Predictors of perceived stigma and social dysfunction

Variable		Perceived stigma		Social dysfunction	
		Median	*P*	%	*P*
Sex	Female	9	0.74	42.0	0.03
	Male	10		25.0	
Employment status	Full time	12	0.001	32.6	0.05
	Part time	7.75		25.0	
	Not working	8.25		29.9	
	Disabled	24		66.7	
Vocal tremor	Present	14.75	0.04	45.5	0.06
	Absent	8.5		29.2	
Head tremor	Present	10	0.52	40.7	0.17
	Absent	10		29.6	
Bigger health concern	Present	10	0.78	35.6	0.74
	Absent	9		32.1	
Social dysfunction	Present	19.5	<0.0001		
	Absent	7			

	Linear regression		Logistic regression	
	coeff (SE)	*P*	OR (SE)	*P*
Current age	−0.28 (0.08)	0.001	0.98 (0.01)	0.14
Age of tremor onset	−0.21 (0.04)	<0.001	0.98 (0.01)	0.015
Duration of tremor	0.14 (0.05)	0.004	1.02 (0.01)	0.083
Stigma psychol distress	0.70 (0.05)	<0.001	1.16 (0.03)	<0.001
Depression	1.18 (0.26)	<0.001	1.52 (0.14)	<0.001
Anxiety	1.18 (0.20)	<0.001	1.34 (0.09)	<0.001
Mindfulness	−0.16 (0.04)	<0.001	0.96 (0.01)	<0.001
Commitment to action	−0.30 (0.11)	0.005	0.92 (0.02)	<0.001
Locus of control	0.45 (0.23)	0.05	1.19 (0.06)	<0.001
Acceptance and action	0.55 (0.09)	<0.001	1.12 (0.03)	<0.001
Cognitive fusion	0.50 (0.08)	<0.001	1.10 (0.03)	<0.001
Resilience	−0.19 (0.07)	0.006	0.95 (0.01)	0.001
Narcissism	−0.18 (0.15)	0.25	0.92 (0.33)	0.029
Self‐monitoring	0.16 (0.26)	0.55	0.96 (0.05)	0.493
Tremor severity	0.45 (0.08)	<0.001	1.09 (0.02)	<0.001
Perceived stigma			1.19 (0.03)	<0.001

*Note*: For categorical independent variables listed in the top half of the table, the median perceived stigma score is shown for each level and statistical test is Wilcoxon ranked sum test, except for employment status analyzed with equality‐of‐medians test. The right side of the table shows % of each category manifesting social dysfunction analyzed with Fisher's exact test. For continuous independent variables listed in the bottom half of this table, the simple regression coefficient (standard error of the estimate) of perceived stigma score is shown, as is the Odds Ratio (standard error of estimate) for presence of social dysfunction.

**TABLE 3:** Associations of tremor treatments with perceived stigma and social dysfunction

	Current	Past	Never	Unknown	*P*
Propranolol: *N*	63	45	44	5	
Perceived stigma score	12	11	4.25	10	0.15
% with social dysfunction	39.7	31.1	27.3	40.0	0.56
Primidone: *N*	31	35	76	15	
Perceived stigma score	11	13	7.25	11	0.05
% with social dysfunction	41.9	48.6	22.4	40.0	0.02
Topiramate: *N*	13	26	105	13	
Perceived stigma score	16.5	15	8.5	10	0.06
% with social dysfunction	53.8	53.8	26.7	30.7	0.02
Deep brain stimulation: *N*	22		135		
Perceived stigma score	12		9		0.18
% with social dysfunction	36.3		33.3		0.80

*Note*: Number of subjects reporting current use or past use of the treatment, or never used, or did not recall if used in the past. For each category, the median perceived stigma score is shown analyzed with equality of medians test, and the % manifesting social dysfunction is shown analyzed with Fisher's exact test.

### Social Dysfunction

53/157 (33.8%) participants met the criterion for social dysfunction. Those who had never used primidone or topiramate were less likely to manifest social dysfunction as compared with current or past users of these medications (Table 3). The clinical‐demographic characteristics predictive of social dysfunction largely overlapped with perceived stigma: worse tremor severity, disabled status, and earlier age of onset; female sex was also associated with this outcome (Table 2). As hypothesized, perceived stigma predicted social dysfunction with Odds Ratio 1.19, *P* < 0.001. Additionally, 10 of the 11 psychological constructs were associated with social dysfunction. In multiple logistic regression perceived stigma (OR 1.2, *P* = 0.002), sex (OR 4.7 for females, *P* = 0.045), stigma psychological distress (OR 1.2, *P* = 0.001) and depression (1.5, *P* = 0.004) were the variables retained, with likelihood ratio chi2(4) = 85 and pseudo *R* ^2^ = 0.62.

## Discussion

The social psychological ramifications of tremor were described by Thangavelu et al as follows: “While performing tasks in a social setting, violation of social rules or norms regarding the expected degree of motor proficiency may give rise to fears regarding negative evaluation, emotions such as shame and guilt, which may lead to embarrassment and avoidance, and increasing levels of functional disability.”^13^ People with ET report that addressing the psychological ramifications of living with ET is one of their greatest unmet needs.^14^ In response, we recently developed a tool to study the stigmatization of people with ET.^2^

Almost all ET patients in this report had perceived ET stigma, especially those with worse tremor severity and with vocal tremor. Age and presence of vocal tremor had significant interaction terms with tremor severity in these models with negative coefficients for each. Negative interaction terms can be viewed as lowering the slopes mapping tremor severity to perceived stigma for older as compared to younger people and for those with vocal tremor as compared to those without. Those with vocal tremor have higher stigma scores independent of tremor severity assessed in terms of manual difficulty; the negative interaction means that when presence of vocal tremor is factored in, worsening manual tremor has less impact on perceived stigma. The negative age interaction may reflect the contexts in which older people find themselves eg, with lesser need to project stability for work or dating or caring less about social judgment as a result of maturity. However half the participants were 70 or older, with only 20% below age 60, and the youngest seven participants aged 30–40 years. It would be interesting to study ET stigma in people younger than this for whom stigma may be worse again.

Identification of factors predisposing to perceived ET stigma has limited therapeutic implications. Suppression of manual or vocal tremor might in theory be expected to reduce ET stigma but tremor suppression cannot always be achieved safely and effectively, and evidence is mixed that psychological welfare is helped even when it is. In our cross‐sectional study, stigma responses were very similar for current and past users of oral medications, and for those with DBS as compared with non‐surgical participants. Participants who had never used tremor medication tended to have less issues with stigma, and this appears to coincide with less severe tremor. A small study assessing efficacy of unilateral thalamic deep brain stimulation reported less anxiety and stigma 3 months later though it was not clear from the study design if this flowed from physical reduction in tremor.^15^ Pharmacologic tremor suppression in another study did not necessarily reduce social embarrassment^16^ suggesting this emotional response can become relatively fixed in some peoples’ cognitive and behavioral patterns due to basic learning processes such as respondent (classical) conditioning.^17^ Holding and Lew^18^ suggested both physical tremor and learned psychological avoidance need be targeted to mitigate ET stigma.

Stigma psychological distress and social dysfunction might be considered downstream consequences of experienced or perceived ET stigma. These relationships and their clinical implications are not well understood. Anecdotally, some people subjected to ET stigma profess to not be troubled and they do not alter their life plans as a result, while others have described a self‐perpetuating cycle of apprehension in anticipation of negative public judgment exacerbating tremor to the extent they have concluded it is better to not eat in public. We have seen one lawyer but not another quit litigation after observers misperceived shakiness as indicative of lack of confidence. Such varied social behavioral responses by people with ET could be due to individual psychological predisposition. In this paper, we evaluated 11 psychological factors that we thought might contribute to stigma social dysfunction even when controlling for perceived stigma.

Here we report that, unsurprisingly, lifetime cumulative perceived ET stigma is predictive of social dysfunction. While effects of age and tremor severity and vocal tremor are encompassed in the perceived stigma effect, females are more likely to manifest social dysfunction when controlling for these other variables. In models relating perceived stigma to social dysfunction, stigma psychological distress and depression were also significant (which echoes a similar finding by Louis et al^19^). These results raise the possibility that interventions aimed at reducing stigma psychological distress and depression might prevent or treat social dysfunction among people who have experienced ET stigma. Cognitive behavioral therapy has potential in this regard as it can lower depression^20^ and improve social avoidance behaviors.^21^ Stigma treatment is an emerging field of study. A theoretic framework is still in evolution, and approaches include attempts to alter beliefs, to enhance coping skills and improve self‐esteem.^22^ Trials of Narrative Enhancement Cognitive Therapy have been undertaken for stigma associated with severe mental illness and results have been mixed, with some^23^, ^24^ but not all^25^ reporting efficacy.

A potential limitation of this study involves its reliance on self‐report. Participant responses may have recall bias and inaccuracy: how reliably do people remember their age at onset of tremor? Do participants mean the same thing when reporting presence vs absence of vocal tremor, or head tremor, which in a prior study was not recognized by almost half those affected.^26^ The response rate (18.3%) was quite low. People unwilling or unable to commit the time necessary to answer the questionnaires or those uncomfortable answering these psychological questions may be under‐represented in our sample. We have reported^2^ that the distributions of age and gender did not differ among participants and non‐participants but are unable to assess other likely self‐selection biases. There is a sizable literature showing that responders to health questionnaires report better health status than non‐respondents, whereas individuals with poorer health or greater stressors tend to avoid participation in health surveys.^27^ Hence, it is possible that we *under‐estimated* the extent of stigmatization and maladaptive behaviors in ET. The targeted study population was all eligible ET patients at a single academic medical center in the southwest United States, which is not necessarily representative of all people with ET. Similar analysis performed in other populations would reveal if our findings can be generalized. Another question that might be addressed is potential contribution to stigma of common ET comorbidities such as gait unsteadiness.

Notwithstanding its limitations, this report clarifies some important associations of perceived ET stigma. It is greater among those with worse tremor severity especially if younger, and in those with vocal tremor. Those who have perceived most tremor stigma manifest more social dysfunction, especially if female; depression and stigma psychological distress are also predictive. Interventions targeting these two psychological constructs might mitigate the social behavioral ramifications among those susceptible to ET stigma.

## Author Roles

(1) Research project: A Conception, B. Organization, C. Execution; (2) Statistical Analysis: A. Design, B. Execution, C. Review and Critique; (3) Manuscript Preparation: A. Writing of first draft, B. Review and Critique.

P.O.: 1B, 1C, 2A, 2B, 3A.

D.S.B.: 2C, 3B.

D.A.L.: 1B, 3B.

T.H.T.: 1B, 3B.

M.T.: 1B, 1C, 3B.

E.D.L.: 1A, 3B.

## Disclosures

**Ethical Compliance Statement:** This study was approved by Institutional Review Board of the University of Texas Southwestern Medical Center. The IRB determined this as minimal risk research, and informed consent was electronic: solicitation was via email of IRB‐approved information about the study, and participant consent was indicated by proceeding to the survey link. All 6 authors have stated “we confirm that we have read the journal's position on issues involved in ethical publication, and affirm that this work is consistent with those guidelines.”

**Funding Sources and Conflicts of Interest:** P.O. was supported by Caroline and Rick O'Brien. E.D.L. was supported by NINDS R01 NS086736. No other funding was received for this work. The authors declare that there are no conflicts of interest relevant to this work.

**Financial Disclosures for the Previous 12 Months:** P.O.S. reports research funding for clinical trials from Abbvie, Biovie, IRLAB and Transposon Therapeutics. T.H.T. reports financial compensation for consultation related to clinical trial design from WCG‐VeraSci (now WCG Clinical Endpoint Solutions) and Scion Neurostim. M.T., D.S.B., and D.A.L. have no funding sources to report. E.D.L. reports research support from the National Institutes of Health: NINDS #R01 NS094607 (principal investigator), NINDS #R01 NS088257 (principal investigator), NINDS #R01 NS117745 (principal investigator), NINDS #R01 NS086736 (principal investigator), and NINDS #R01 NS124854 (co‐investigator).

## References

[1] Shalaby S , Indes J , Keung B , et al. Public knowledge and attitude toward essential tremor: a questionnaire survey. Front Neurol 2016;7:60. 27148160 10.3389/fneur.2016.00060 PMC4840213

[2] O'Suilleabhain PE , Tovar M , Wagle Shukla A , Tester N , Lundervold DA , Turner TH , et al. Development of ETStig, a measure for stigma in essential tremor. Parkinsonism Relat Disord 2022;104:38–43. 36215750 10.1016/j.parkreldis.2022.09.009

[3] Lundervold DA , Ament PA , Holt PS . RE‐examination of the tremor disability scale‐revised (TREDS‐R): a case control comparison. N Am J Psychol 2018;20(2):277–287.

[4] Baer RA , Smith GT , Hopkins J , Krietemeyer J , Toney L . Using self‐report assessment methods to explore facets of mindfulness. Assessment 2006;13(1):27–45. 16443717 10.1177/1073191105283504

[5] Zigmond AS , Snaith RP . The hospital anxiety and depression scale. Acta Psychiatr Scand 1983;67(6):361–370. 6880820 10.1111/j.1600-0447.1983.tb09716.x

[6] Gillanders DT , Bolderston H , Bond FW , et al. The development and initial validation of the cognitive fusion questionnaire. Behav Ther 2014;45(1):83–101. 24411117 10.1016/j.beth.2013.09.001

[7] Bond FW , Hayes SC , Baer RA , et al. Preliminary psychometric properties of the acceptance and action questionnaire‐II: a revised measure of psychological inflexibility and experiential avoidance. Behav Ther 2011;42(4):676–688. 22035996 10.1016/j.beth.2011.03.007

[8] McCracken LM , Chilcot J , Norton S . Further development in the assessment of psychological flexibility: a shortened committed action questionnaire (CAQ‐8). Eur J Pain 2015;19(5):677–685. 25181605 10.1002/ejp.589

[9] Rotter JB . Generalized expectancies for internal versus external control of reinforcement. Psychol Monogr Gen Appl 1966;80(1):1–28. 5340840

[10] Connor KM , Davidson JR . Development of a new resilience scale: the Connor‐Davidson resilience scale (CD‐RISC). Depress Anxiety 2003;18(2):76–82. 12964174 10.1002/da.10113

[11] Raskin R , Terry H . A principal‐components analysis of the narcissistic personality inventory and further evidence of its construct validity. J Pers Soc Psychol 1988;54(5):890–902. 3379585 10.1037//0022-3514.54.5.890

[12] Snyder M . Self‐monitoring of expressive behavior. J Pers Soc Psychol 1974;30(4):526–537.

[13] Thangavelu K , Talk AC , Clark GI , Dissanayaka NNW . Psychosocial factors and perceived tremor disability in essential tremor. Neurosci Biobehav Rev 2020;108:246–253. 31682885 10.1016/j.neubiorev.2019.10.021

[14] Louis ED , Rohl B , Rice C . Defining the treatment gap: what essential tremor patients want that they are not getting. Tremor Other Hyperkinet Mov (N Y) 2015;5:331. 26317044 10.7916/D87080M9 PMC4548969

[15] Troster AI , Fields JA , Pahwa R , Wilkinson SB , Strait‐Troster KA , Lyons K , et al. Neuropsychological and quality of life outcome after thalamic stimulation for essential tremor. Neurology 1999;53(8):1774–1780. 10563627 10.1212/wnl.53.8.1774

[16] Koller W , Biary N , Cone S . Disability in essential tremor: effect of treatment. Neurology 1986;36(7):1001–1004. 2940473 10.1212/wnl.36.7.1001

[17] Greco JA , Liberzon I . Neuroimaging of fear‐associated learning. Neuropsychopharmacology 2016;41(1):320–334. 26294108 10.1038/npp.2015.255 PMC4677141

[18] Holding SJ , Lew AR . Relations between psychological avoidance, symptom severity and embarrassment in essential tremor. Chronic Illn 2015;11(1):69–71. 25053752 10.1177/1742395314544554

[19] Louis ED , Cosentino S , Huey ED . Depressive symptoms can amplify embarrassment in essential tremor. J Clin Mov Disord 2016;3:11. 27429787 10.1186/s40734-016-0039-6 PMC4947359

[20] Sudak DM . Cognitive behavioral therapy for depression. Psychiatr Clin North Am 2012;35(1):99–110. 22370493 10.1016/j.psc.2011.10.001

[21] Leichsenring F , Salzer S , Beutel ME , et al. Long‐term outcome of psychodynamic therapy and cognitive‐behavioral therapy in social anxiety disorder. Am J Psychiatry 2014;171(10):1074–1082. 25016974 10.1176/appi.ajp.2014.13111514

[22] Mittal D , Sullivan G , Chekuri L , Allee E , Corrigan PW . Empirical studies of self‐stigma reduction strategies: a critical review of the literature. Psychiatr Serv 2012;63(10):974–981. 22855130 10.1176/appi.ps.201100459

[23] Hansson L , Lexen A , Holmen J . The effectiveness of narrative enhancement and cognitive therapy: a randomized controlled study of a self‐stigma intervention. Soc Psychiatry Psychiatr Epidemiol 2017;52(11):1415–1423. 28424854 10.1007/s00127-017-1385-x PMC5663807

[24] Oudejans S , de Winter L , van Weeghel J , Sanches S , Hasson‐Ohayon I . Feasibility and outcomes of narrative enhancement and cognitive therapy (NECT) for reducing self‐stigma among people with severe mental illness in The Netherlands: a pilot study. Psychiatr Rehabil J 2022;45:255–265. 35913854 10.1037/prj0000526

[25] Yanos PT , Roe D , West ML , Smith SM , Lysaker PH . Group‐based treatment for internalized stigma among persons with severe mental illness: findings from a randomized controlled trial. Psychol Serv 2012;9(3):248–258. 22545821 10.1037/a0028048 PMC3413784

[26] Eken HN , Louis ED . Agnosia for head tremor in essential tremor: prevalence and clinical correlates. J Clin Mov Disord 2016;3:4. 26877883 10.1186/s40734-016-0032-0 PMC4751737

[27] Cheung KL , Ten Klooster PM , Smit C , de Vries H , Pieterse ME . The impact of non‐response bias due to sampling in public health studies: a comparison of voluntary versus mandatory recruitment in a Dutch national survey on adolescent health. BMC Public Health 2017;17(1):276. 28330465 10.1186/s12889-017-4189-8 PMC5363011

